# Low Self-Esteem and Its Association With Anxiety, Depression, and Suicidal Ideation in Vietnamese Secondary School Students: A Cross-Sectional Study

**DOI:** 10.3389/fpsyt.2019.00698

**Published:** 2019-09-27

**Authors:** Dat Tan Nguyen, E. Pamela Wright, Christine Dedding, Tam Thi Pham, Joske Bunders

**Affiliations:** ^1^Faculty of Public Health - Can Tho University of Medicine and Pharmacy, Can Tho City, Vietnam; ^2^Guelph International Health Consulting, Amsterdam, Netherlands; ^3^VU University Athena Institute, Faculty of Earth and Life Sciences, Vrije Universiteit Amsterdam, Amsterdam, Netherlands

**Keywords:** self-esteem, anxiety, depression, suicide, adolescents, Vietnam

## Abstract

**Background:** There is a correlation between self-esteem in adolescents and risks and protective factors for their health and welfare. The study was conducted to determine the prevalence of low self-esteem and sociodemographic features related to anxiety, depression, educational stress, and suicidal ideation in secondary school students in Vietnam.

**Methods:** A cross-sectional design was employed for this study with participation of 1,149 students in Cantho City in Vietnam. A structured questionnaire was applied to ask about self-esteem, depression, anxiety, educational stress, and suicidal ideation.

**Results:** Students with low self-esteem were detected at a prevalence of 19.4%. High educational stress and physical and emotional abuse by parents or other adults in the household were major risk factors correlated to low self-esteem, while a protective factor for low self-esteem was attending supplementary classes. An association among lower self-esteem and increased anxiety, depression, and suicidal ideation was detected.

**Conclusions:** Self-esteem is associated with anxiety, depression, and academic stress, which significantly affect students’ quality of life and links to suicidal ideation. These results therefore suggested the need for a school-based or web-based provision aimed at proactively increasing students’ self-esteem and skills for dealing with academic stress.

## Introduction

It is well recognised that adolescence is one of the most rapid phases of human development (1). It is characterised by a rapid physical, social, and cognitive growth, as well as changes in self-esteem. Self-esteem is reported to have a significant impact on important life outcomes including health and social outcomes during adolescence and adulthood. For example, there is a clear connection between higher self-esteem and positive outcomes, such as occupational success, better social relationships, a sense of well-being, and positive perceptions by peers, academic achievement, and good coping skills (2, 3). Low self-esteem is causally related to depression, substance abuse, antisocial behaviour, and suicide (4–6). The literature demonstrates that social functioning, such as acceptance by peers, is lower in children with low self-esteem (7).

Extensive research has explored risk and protective factors related to low self-esteem development during adolescence. Reported risk factors include being a girl (6, 8, 9), the family’s low socioeconomic status (10), parents’ education level, family eligibility for public assistance, eligibility for free or reduced-cost school meals, the parents’ employment status (11), and school performance and grades (6, 12), as well as obesity (13).

Academic achievement is known to be affected by self-esteem, while self-esteem may also influence academic achievement (7). High self-esteem is reported as an important predictive factor for students’ academic achievement (14). Another study found that while high self-esteem resulted in many positive outcomes and benefits, it did not necessarily lead to good school performance (15). On the other hand, adolescents with poor academic results did not always have low general self-esteem (16).

There is an association between low self-esteem and negative outcomes for young people’s behavioural and mental health problems, including health-compromising behaviours such as substance abuse, early sexual activity, and eating problems (17). A longitudinal study among a large sample of young New Zealanders found, however, that while low self-esteem significantly predicted adolescent eating and other health-compromising behaviours, it was not related to substance abuse and early sexual activity (18). With regard to mental health, a correlation has been detected between low self-esteem and depression (19, 20), anxiety (10, 21), and adolescents’ suicidal ideation and attempts (18, 22).

Reports during the past few years indicate that there is a strong relationship between academic pressure and stress, depression, anxiety, low self-esteem, and suicidal ideation among students in secondary or high school and in young adults (23–25). A study describing perceptions about mental illness in Hue in Vietnam noted that “studying or thinking too much” is a cause of mental health problems (26). In Vietnamese culture, the pressure from parents and schools might be expected to have an unintended effect, leading to lower self-esteem and associated outcomes seen elsewhere among high school students. There are as yet no reports of systematic studies on this issue in Vietnam, and there are few reports of descriptive or analytical research into adolescent self-esteem.

This is particularly relevant since we found high levels of depression, anxiety, and suicidal ideation among secondary school students in Vietnam [Nguyen, D. T., Dedding, C., Pham, T. T., Wright, P., & Bunders, J. (25)]. This article reports on a study that aimed to determine (1) the prevalence of low self-esteem; (2) the characteristics associated with low self-esteem; and (3) the relationships among self-esteem and anxiety, depression, educational stress, and suicidal ideation in secondary school students. We hypothesised that low self-esteem would be associated with a greater risk of poor mental health status.

## Materials and Methods

### Study Design and Participants

A cross-sectional study design was used to recruit 1,260 students at three secondary schools in urban and suburban areas in Cantho City in Vietnam. All data were collected during the first academic semester, from September to December 2011. A more detailed description of the sampling and its participants can be found elsewhere (25).

### Measures

All participants were invited to provide information by self-reporting using a questionnaire. This was done after class or at home anonymously to minimise potential reporting bias and to keep information confidential. The detailed components of the questionnaire have been described elsewhere (25).

The 10-item Rosenberg Self-esteem Scale (21, 27) was used to assess global self-esteem, with higher scores indicating more positive self-regard. Each item asked for response using a 4-point Likert scale ranging from 1 (strongly agree) to 4 (strongly disagree). The scale is generally reliable, with test–retest correlations value between 0.82 and 0.88 (21). The Cronbach α of the scale in the present study was 0.77. The scale ranges from 0 to 30: a score greater than 25 suggests high self-esteem; scores between 15 and 25 are considered to be within normal range, whereas scores less than 15 suggest low self-esteem (Morris Rosenberg).

A more detailed method description on employing the Center for Epidemiology Studies Depression (CES-D), the anxiety scale, and the Educational Stress Scale for Adolescents for this study is available (25).

To address the issue of suicidal ideation, additional questions on whether the student had ever seriously considered suicide or made a suicide plan used a 3-point scale (never, sometimes, and often). A yes/no question was also used to identify students who had attempted suicide.

To define risks and protective factors, we explore the variables listed in **Table 1**, including mother’s education, physical and emotional abuse by parents or other adults in the household or school, academic performance in the last semester, educational stress, attendance at a supplementary class, and use of personal tutor. The data collected using the questionnaire is available in the **Supplementary Data File**.

**Table 1:** Frequencies and percentages (%) of sociodemographic variables of participants by self-esteem status.

	Total		Low self-esteem		Normal self-esteem		*p* ^a^
	n	%	N	%	n	%	
Total screened	1,149	100	223	19.4	926	80.6	
**Age, mean (SD)**	16.05		16.01		16.05		0.290
**Sex**			223		926		0.085
Male	419	36.5	72	32.3	347	37.5	
Female	730	63.5	151	67.7	579	62.5	
**Family economic status**	1,148		222		926		<0.001
Comfortable living and wealthy	664	57.8	100	45,0	564	60,9	
Very poor/poor/earn just enough to live	484	42.2	122	55.0	362	39.1	
**Academic performance in the last semester**	1,130		219		911		0.003
Excellent/good	225	19.9	33	15.1	192	21.1	
Fairly good/average	854	75.6	168	76.7	686	75.3	
Below average/very poor	51	4.5	18	8.2	33	3.6	
**Personal tutor**	1,142		223		919		0.004
Yes	928	81.3	166	74.4	762	82.9	
No	214	18.7	57	25.6	157	17.1	
**Attendance at supplementary class**	1,146		223		923		0.003
None	323	28.2	81	36.3	242	26.2	
One or more	823	71.8	142	63.7	681	73.8	
**Mother’s education**	1,143		222		921		0.001
> Primary school	1,006	88.0	181	81.5	825	89.6	
<= Primary school	137	12.0	41	18.5	96	10.4	

^a^ *χ* ^2^ was used to compare differences in percentages; t test was used to compare differences in means.

### Data Analysis

Demographic data were analysed descriptively to determine basic characteristics of the sample population and presented as means ± standard deviations (SD). The χ^2^ test was used to assess the significance of differences in the distribution of participants by selected sociodemographic characteristics, risk factors, and outcome variables. Associations between low self-esteem and family characteristics, educational stress, and academic achievement were explored by logistic regression analysis. Univariate independent predictors of low self-esteem with *p* < 0.10 were entered in a multivariate logistic regression model, and the backward Wald method was applied to study their influence on the presence of low self-esteem. Univariate logistical analyses were also applied to determine the relationships among low self-esteem and anxiety, depression, and suicidal ideation, and Pearson correlation coefficients were used to measure the correlations among these. All analyses were carried out using a significance level of 5%, and all tests were 2-sided. The 95% confidence intervals (CIs) of odds ratios (ORs) were also calculated. All analyses were analysed by using SPSS Inc, Chicago, the United States of America version 16.0.

## Results

### Demographics

Of the 1,260 students invited to participate, 111 (7.3%) were excluded from analysis because they did not adequately complete the questionnaire; for example, they did not provide answers to five or more items in the CES-D. The final sample comprised 1,149 senior high school students with a mean age of 16.1 years. Participating students were fairly evenly distributed in terms of sex: 36.5% males and 63.5% females (**Table 1**). This proportion also reflects the proportion of males and females enrolled in the classes studied. Students’ participation from each grade (10–12) did not significantly differ, at around 33% each. Ninety-five percent of students were ethnically Kinh; other ethnic groups included Hoa and Khmer. This sample was in line with the school population in this area.

### Prevalence of Low Self-Esteem and Characteristics Associated With Self-Esteem

The mean self-esteem score was 17.56 on a scale of 0 to 30. The scores for boys and girls were similar (17.83 vs. 17.40; *t* = 1.720; *p* = 0.086). Based on all scores, nearly a fifth (19.4%) of the respondents reported low self-esteem according to Rosenberg’s criteria, with scores below 15 (**Table 1**). Based on univariate logistic regression analysis, among six investigated variables, two variables “having a personal tutor” and “attendance at supplementary class” were negatively associated with the risk of low self-esteem, while the other variables were positively correlated to low self-esteem (**Table 2**). Students were likely to show lower self-esteem, when their mother’s education was at primary level or below, when they were often physically or emotionally abused at home or at school, when they had below average/very poor academic performance in the last semester, or when they reported experiencing high educational stress.

**Table 2:** Factors associated with low self-esteem: multivariate logistic regression analyses.

**Factors**	**Low self-esteem** **n (%)**	**Normal self-esteem** **n (%)**	**Univariate logistic regression**		**Multivariate logistic regression**	
			***OR (95% CI)***	***p-value***	***OR (95% CI)***	***p-value***
**Mother’s education (n = 1,143)**						
> Primary school	181 (18.0)	825 (82.0)	Reference	–	Reference	–
≤ Primary school	41 (29.9)	96 (90.1)	**1.95 (1.31–2.90)**	0.001	**1.68 (1.09–2.69)**	**0.018**
**Physical and emotional abuse by parents or other adults in the household (n = 1,146)**						
No	192 (18.5)	845 (81.5)	Reference	–	Reference	–
Yes	31 (28.4)	78 (71.6)	**1.75 (1.12–2.73)**	0.013	**1.76 (1.10–2.82)**	**0.018**
**Academic performance in the last semester (n = 1,130)**						
Excellent/good	33 (14.7)	192 (85.3)	Reference	–	Reference	–
Fairly good/average	168 (19.7)	686 (80.3)	1.43 (0.95–2.14)	0.087	–	–
Below average/very poor	18 (35.5)	33 (64.7)	**3.17 (1.60–6.28)**	0.001	–	–
**Educational stress (n = 1,142)**						
Low stress	31 (9.4)	300 (90.6)	Reference	–	Reference	–
Medium stress	68 (17.4)	322 (82.6)	**2.04 (1.30–3.22)**	**0.002**	**2.25 (1.41–3.58)**	**0.001**
High stress	121 (28.7)	300 (71.3)	**3.90 (2.55–5.98)**	**<0.001**	**4.02 (2.60–6.23)**	**<0.001**
**Personal tutor (n = 1,142)**						
Yes	166 (17.9)	762 (82.1)	Reference	–	–	–
No	57 (26.6)	157 (73.4)	**1,67 (1,18–2,36)**	**0,004**	–	–
**Attendance at supplementary class (n = 1,146)**						
None	81 (25.1)	242 (74.9)	Reference	–	Reference	–
One or more	142 (17.3)	681 (82.7)	**0.62 (0.46–0.85)**	**0.003**	**0.57 (0.41–0.79)**	**0.001**
**Total (n = 1149)**	**223 (19.4)**	**926 (80.6)**				

Significance levels were at the 0.05 level (2-tailed).

In the multivariate regression analysis (with the backward Wald method), not accounting for effect modification, four variables remained correlated to low self-esteem, either in a negative or positive direction. Having a personal tutor and academic performance in the last semester were no longer a significant association, but attending supplementary classes still remained a protective factor for low self-esteem (reduced OR of 43% compared with students not attending) (**Table 2**).Study results that were considerably positively associated to low self-esteem were medium and high educational stress (ORs = 2.25 and 4.02, respectively).

### Relationships Between Self-Esteem and Anxiety, Depression, and Educational Stress, and Suicidal Ideation

As shown in **Table 3**, four variables—self-esteem, anxiety, depression, and educational stress—were related to each other. Self-esteem was negatively correlated to anxiety, depression, and educational stress, while educational stress was positively correlated to anxiety and depression.

**Table 3:** Pearson correlations between self-esteem, anxiety, depression, and educational stress.

	Self-esteem	Anxiety	Depression	Educational stress
**Self-esteem**	–			
**Anxiety**	−0.219	–		
**Depression**	−0.426	0.422	–	
**Educational stress**	−0.249	0.318	0.308	–

*All correlations are significant at the 0.001 level (2-tailed).*

One of the purposes of the study was to identify the impact of self-esteem on mental health problems. The results of univariate logistics indicate that low self-esteem contributed significantly to anxiety, depression, and suicide among adolescents. Compared to students who reported normal self-esteem, the students who reported low self-esteem had twice the odds of having anxiety symptoms [20.3% (187/921) vs. 34.2% (76/222)] (OR = 2.04; 95% CI OR = 1.48–2.82, *p* < 0.001), nearly six times the odds of being at risk of depression (CES-D score of ≥16) (74.0% vs. 33.2%), (OR = 5.72; 95% CI OR = 4.11–7.59), four times the odds of having depressive symptoms (CES-D scores of > 21) (50.7% vs. 19.8%) (OR = 4.16; 95% CI OR = 3.06–5.66), and nearly five times the odds of having depression (CES-D score of > 25) (41.7% vs. 13.0%), (OR = 4.79; 95% CI OR = 3.45 – 6.65). Students with low self-esteem also were significantly more likely to have considered or attempted suicide (**Table 4**).

**Table 4:** Associations between self-esteem and suicidal ideation.

	Yes n (%)	No n (%)	OR (CI 95%)	p
**Seriously considered suicide**	***301 (26.3)***	***844 (73.7)***		
Normal self-esteem	213 (23.1)	710 (76.9)	–	–
Low self-esteem	88 (39.6)	134 (60.4)	**2.19 (1.61–2.98)**	**<0.001**
**Planning suicide**	***142 (12.8)***	***965 (87.2)***		
Normal self-esteem	96 (10.8)	792 (89.2)	–	–
Low self-esteem	46 (21.0)	173 (79.0)	**2.19 (1.49–3.23)**	**<0.001**
Attempted suicide	44 (3.9)	1,095 (96.1)		
Normal self-esteem	28 (3.1)	884 (96.9)	–	–
Low self-esteem	16 (7.3)	204 (92.7)	**2.48 (1.32–4.66)**	**0.004**

*Significance levels were at the 0.05 level (2-tailed).*

## Discussion

We investigated the prevalence of low self-esteem among secondary school students, the characteristics associated with low self-esteem, and the relationships among self-esteem and anxiety, depression, and suicide. Nearly 20% of the students had low self-esteem, with no difference between girls and boys. These results are in line with some previous studies (12, 28), but inconsistent with others (6, 12). Cultural and social differences in the study populations could explain some of the differences.

### Reasons for Having a Higher Prevalence of Low Self-Esteem Among Females

The results of this study cannot provide precise reasons for the higher prevalence of lower self-esteem among females; however, a number of possible causes for the gender differences are worth discussing. Although gender equality has improved in recent years in Vietnam and is clearly legislated, gender issues within the family and society remain a challenge for most Vietnamese, who are strongly influenced by traditional culture and custom (29). There is an association between female gender and emotionally unstable personality (30, 31). As a result, women are more prone to develop emotionally unstable personality (e.g. borderline personality) (32). Thus, females’ self-esteem may be more likely to fluctuate, depending on how they themselves or others, especially their parents, evaluate their achievements.

A high prevalence of low self-esteem among the secondary school students should be considered as an important mental health problem not only by parents, school teachers, and principals but also by policy makers in the education and health sectors in Vietnam. In fact, health care related to psychological disorders has not received adequate consideration yet in Vietnam (33). Therefore, it is important to take mental health care into account when developing a policy framework to improve general school health services, such as directive no. 23/2006/CT-TTg on having well-equipped and professionally qualified health officers in schools (34), and Decision No. 401/2009/QD-TTg, which approved the program “Preventing and combatting diseases in educational establishments belonging to the national education system” (35).

Our results confirm the findings of previous studies (6, 10, 25, 36), namely, that family characteristics, including mother’s low educational level and physical and emotional abuse by parents or other adults in the household, were associated with low levels of self-esteem. In addition, educational characteristics such as the school environment, academic performance, and high educational stress were strongly associated with self-esteem. These findings are consistent with results of other studies, for example, by Pullmann & Allik (16), which found that parents have a great deal of influence on their children’s psychological development, including on the relationships outside the family environment (6, 10). Students who attended one or more supplementary classes appeared to be at lower risk of having poor self-esteem. In Vietnam, these students are usually from a family with a higher socioeconomic status. Attending supplementary class may reduce the stress of workload and academic pressure for those students. It would be useful to confirm this with further studies in the Vietnamese context, but this kind of support is unlikely to be available to families who cannot pay for it.

Low self-esteem in the adolescents in Cantho City was associated with poor academic performance. Aryana (14) reported that high self-esteem among preuniversity students was an important factor in predicting high academic achievement. Our finding also highlights that self-esteem plays an important role in predicting academic achievement, although a cross-sectional study design is unable to demonstrate causality; a prospective study to identify causality of the low self-esteem would be appropriate in future research.

With regard to mental health, the results of Pearson correlation analysis showed that self-esteem was negatively correlated to anxiety, depression, and educational stress, while the results of univariate logistic regression illustrated that low self-esteem contributed to a high risk of anxiety, depression, and suicidal ideation. These results are comparable to others showing that low self-esteem was associated with depression (19, 20, 37) and identifying a relationship between self-esteem and anxiety (10, 21), as well as a relationship between low self-esteem and suicidal ideation and suicide attempts among adolescents (18, 22). Screening for low self-esteem in adolescents is a possible strategy to help identify secondary school students at risk of anxiety, depression, and suicide. In addition, our previous study (25) showed an association between mental health problems and academic pressure, resulting from an overloaded curriculum and pressure from teachers and parents to succeed. There is clearly a need to reduce mental health problems among today’s secondary school students in Vietnam. The development of a website to provide psychoeducation designed to meet the needs of young Vietnamese and of school-based counselling services for students, possibly by collaborating with volunteers from the Youth Union, the largest sociopolitical Vietnamese youth organization, at local universities, teachers, and parents, will be explored as one of the solutions (36). Around 66.3% of young Vietnamese downloaded the mobile health applications for disease prevention (38). Nevertheless, one of the challenges to using a digital health intervention is that young Vietnamese with a higher perceived stress level were significantly less likely to use such interventions (39).

### Study Limitations

One limitation of this study relates to the sample and its generalizability. Although the distribution of girls and boys reflected the true/current situation in the classes selected, the sample was from only three schools in urban and suburban Cantho. Another limitation concerns the study design, which specified collection of data from adolescents by self-reporting using standardised questionnaires. The respondents’ personality and identity development are still incomplete, which could result in fluctuating self-perceptions (9) and thus unreliable reporting. Also, the exploration of potential causes of low self-esteem as self-reported by subjects was not included in the survey. Another limitation is that the cross-sectional study design does not permit detection of links between covariates; longitudinal studies on this topic are still needed. Finally, the self-esteem, anxiety, and CES-D scales, like other screening instruments, cannot be viewed as diagnostic tools, but only as screening tests to identify members of groups at risk for these conditions. The results tell us how the students perceive their health but are not in themselves evidence of medical concerns. Moreover, there is no assessment of stress coping and stress levels, obesity (40), and chronic medical illness (e.g. asthma) (41), which may confound and influence anxiety, depression, and suicidal ideations. Finally, in a cross-sectional study, the cause–effect relationship cannot be measured; that requires a longitudinal cohort or a randomised controlled study.

## Conclusions

Our research suggested that self-esteem is correlated to anxiety, depression, and academic stress, which significantly affects students’ quality of life and is linked to suicidal ideation. The results therefore suggest the need for a school-based or web-based provision of information or services aimed at proactively increasing students’ self-esteem and skills for dealing with academic stress.

## Ethics Statement

The study was approved by the Scientific and Training Committees of Cantho University of Medicine and Pharmacy. Written informed consent for participation was obtained from students’ parents or legal guardians before data collection.

## Author Contributions

A first draft of the manuscript was prepared by DN under the guidance of CD. All authors contributed to the final manuscript version. DN, JB, and CD jointly generated the research idea and the study design. DN, CD, TP, and EW contributed to the survey tools. DN and TP coordinated the surveys and data collection in the field. DN, TP, CD, and EW carried out data analysis.

## Funding

The study has been supported by a scholarship from the “Strengthening teaching and research capacity of preventive medicine staffs to meet the challenges of emerging infections and new environmental hazards to health” Project, collaboration between Hanoi University, NUFFIC, and the Dutch Government.

## Conflict of Interest

The authors declare that the research was conducted in the absence of any commercial or financial relationships that could be construed as a potential conflict of interest.
